# Direct non-medical costs double the total direct costs to patients undergoing cataract surgery in Zamfara state, Northern Nigeria: a case series

**DOI:** 10.1186/s12913-015-0831-2

**Published:** 2015-04-16

**Authors:** Nazaradden Ibrahim, Francisco Pozo-Martin, Clare Gilbert

**Affiliations:** Zamfara State Eye Care Programme, Ministry of Health, Gusau, Nigeria; Department of Clinical Research, London School of Hygiene & Tropical Medicine, London, UK; International Centre for Eye Health, Department of Clinical Research, London School of Hygiene & Tropical Medicine, Keppel Street, WC1E 7HT London, UK

**Keywords:** Direct medical costs, Out of pocket expenditure, Cataract surgery, Nigeria

## Abstract

**Background:**

Cost is frequently reported as a barrier to cataract surgery, but few studies have reported costs of accessing surgery in Africa. The purpose of this prospective, facility based study was to compare direct non-medical cost with total direct cost of cataract surgery to patients, and to assess how money was found to cover costs.

**Methods:**

Participants were those aged 17 years and above attending their first post-operative visit after first eye, subsidised, day case cataract surgery. Systematic random sampling was used to select participants who were interviewed to obtain data on socio-demographic details, and on expenditure during the assessment visit, the surgical visit, and the first follow-up visit. Costs were a) direct medical costs (patients’ costs for registration, investigations, surgery, medication), and b) direct non-medical costs (patients’ and escorts’ costs for transport, accommodation, meals). The source of funds to pay for the services received was also assessed.

**Results:**

Almost two thirds (63%) of the 104 participants were men. The mean age of men was 64 (±12.5) years, being 63 (±12.9) years for women. All men were married and 35% of women were widows. 84% of men were household heads compared with 6% of women. The median total direct cost for all visits by all participants was N8,245 (US$51), being higher for men than women (N9,020; US$56 and N7,620; US$47) (p < 0.09) respectively. Direct non-medical cost constituted 49% of total direct cost. 92% of participants had adequate money to pay, but 8% had to sell possessions to raise the money. 20% of unmarried women sold possessions or took out a loan.

**Conclusion:**

Despite the subsidy, cost is still likely to be a barrier to accessing cataract surgery, as the total direct costs represented at least 50 days income for 70% of the local population. Provision of transport would reduce direct non-medical costs.

## Background

Cataract is responsible for blindness in more than 20 million people [1] and may reach 50 million by 2020 [2]. In Nigeria cataract is the leading cause of blindness (43%), affecting an estimated 400,000 adults [3] which is projected to reach over 570,000 by 2020 [4]. However, in 2008 the cataract surgical rate (CSR) in Nigeria was only 300/million population/year, significantly below that recommended for Africa i.e. 2000 [5]. Cataract surgical coverage (<3/60 at the person level) is also low at 38% [6]. Cost is a frequently quoted barrier to cataract surgery, including Nigeria [6,7].

Patient costs include direct and indirect costs. Direct costs comprise direct medical costs (fees charged to patients) and direct non-medical costs (additional costs in accessing treatment e.g. for travel, accommodation, meals) [8]. Indirect costs are the earnings lost by patients and their carers while seeking medical care [8]. In Africa patients are usually accompanied by the head of household or others responsible for payment which increases direct non-medical costs. Direct medical costs for a particular service are likely to be relatively stable but direct non-medical costs can vary considerably. There are several ways in which eye care providers can address cost as a barrier, including national health insurance, provision of transport, outreach surgical camps, cross subsidy and external support, from donors, for example.

Health expenditure is “catastrophic” when it exceeds some proportion of household income or household expenditure, although there is no consensus concerning what proportion [9]. Household’s capacity to pay, and the consequences of raising/finding the money can also be assessed [10]. For example, a study in India defined catastrophic expenditure as “that which reduces non-health expenditure to a level where the household is unable to maintain consumption of necessities” [11]. In Bangladesh, the mean expenditure for children hospitalization with pneumonia was almost US$100, being more than half the total monthly household expenditure for 75% of families [12]. Three quarters of these families raised money through borrowing or selling assets and half planned to reduce spending on food and education for their children. Catastrophic expenditure does not, therefore, imply that costs are excessively high, as even a relatively low cost may constitute a high proportion of household income of the poor. There is a large body of literature in other areas of health on the impact of user fees (i.e. direct costs) on access to services, and many countries have removed user fees for selected services, such as child health and maternal health [13]. The impact of removing user fees is complex, but many studies show a positive impact on access and outcomes but the benefits are not always maintained over time [14]. Although several studies have assessed willingness to pay for cataract surgery i.e. in China, Nepal, Tanzania, to our knowledge only one study has addressed direct patient costs for cataract surgery, in Zambia [15].

In Zamfara State 40% of the 3.6 million population live in the state capital, Gusau. The rural population are mainly subsistence farmers and 70% live on less than a dollar a day [16]. The road network is poor with very limited public transport. Gusau Eye Clinic, which is one of two eye clinics providing cataract surgery in Zamfara state [16], has three ophthalmologists who perform small incision day case cataract surgery. Cataract surgery is performed twice a week on approximately 50 patients and follow up clinics are also held twice a week for approximately 100 patients with a 95% attendance. In 2013, 2,368 cataract operations were performed, 46% on women. However, the unit is not functioning at capacity and there is no waiting list for cataract surgery. An international non-government organization supports the cataract service by providing consumables, spectacles and post-operative medication. They also donated equipment, renovated the infrastructure and trained personnel. This support enables cataract services to be subsidised, and patients contribute N3,000 ($18.5) towards costs of surgery. Without this subsidy costs would be N5,500 ($34) higher. Patients with operable cataract make at least three visits, for initial assessment, for surgery a few weeks later, and follow-up at two weeks. Most are accompanied by an escort. The aim of this study was to compare the direct non-medical cost of cataract surgery with the total direct costs (i.e. medical and non-medical costs) and to investigate how patients raised the money.

## Methods

Every third person attending their first post-operative visit was systematically selected. All had paid the standard fee for cataract surgery on their first operated eye.

Assuming that approximately 20 postoperative patients would be eligible each clinic day, this would give 200 eligible participants over the 5 weeks of the study. A sample of this size was considered adequate, giving a reasonably representative sample and an indication of the range of costs incurred.

Eligible participants had to be aged 17 years and above and 1) have undergone recent conventional cataract surgery on their first eye 2) be blind in the other un-operated eye (i.e. presenting visual acuity of <3/60) from any cause and 3) attending within 2-4 weeks of cataract surgery.

Participants were interviewed, after obtaining written informed consent, with their escort, if present. Two ophthalmic nurses bilingual in English and Hausa conducted the interviews after two days of training. Data were collected using a structured questionnaire on age, sex, marital and socio-economic status e.g. educational level, relationship to head of household, household size, type of sanitation and wealth assessment i.e. ownership of farm/land, type of housing, and asset ownership such as radio or television. The data collection instrument was pre-tested on postoperative patients who were not included in the study.

For each visit the following information was obtained: number of escorts, number of meals away from home, mode of transport and accommodation and their costs. Costs of hospital registration, surgery, investigations, medication and other charges were also assessed for each visit. Participants were asked how they found the money e.g. already available, or if they had to borrow money or mortgage or sell assets.

Data were entered into a Microsoft Access database and analysed in STATA 13.0. Parametric analysis was performed for normally distributed data, and non-parametric tests for skewed data (e.g. costs). Significant levels are reported at p < 0.05. Total direct patient costs were calculated and the proportion due to direct non-medical costs determined. Analysis was conducted using Nigerian Naira (N) at the exchange rate of US$1 to N161.

Ethical approval was obtained from the institutional review board/ethics committee of the London School of Hygiene & Tropical Medicine and the Ministry of Health, Zamfara state. The study adhered to the tenets of the Helsinki Declaration.

## Results

104 participants were interviewed, 66 (63%) of whom were female (Table 1). The mean age of men was 64 years (standard deviation ±12.5 years) being 63 ± 12.9 years for women. The majority (95%) had not received any formal education. All men were married, but 35% of women were widows (p < 0.001). A high proportion of men (84%) were head of their households and 16% were sons of household heads. Almost two thirds (59.1%) of women were daughters of household heads and only four (6%) were household heads themselves (p < 0.001). Most households were large, with ten or more members.

**Table 1:** **Demographic distribution of study participants**

**Variables**		**Male**		**Female**		**All**	
		**N**	**%**	**N**	**%**	**N**	**%**
Age group	30-39 years	1	2.6	2	3.0	3	2.9
	40-49 years	1	2.6	4	6.1	5	4.8
	50-59 years	10	26.3	12	18.2	22	21.2
	60-69 years	11	28.9	27	40.9	38	36.5
	70 years and above	15	39.5	21	31.8	36	34.6
	Total	38	100	66	100	104	100
Educational level	Above secondary	2	5.3	0	0	2	1.9
	Secondary	0	0	2	3	2	1.9
	Primary	0	0	1	1.5	1	1
	Informal	36	94.7	63	95.5	99	95.2
	None	0	0	0	0	0	0
	Total	38	100	66	100	104	100
Marital status	Married	38	100	41	62.1	79	76
	Widow/widower	0	0	23	34.9	23	22.1
	Divorced	0	0	2	3	2	1.9
	Single/never married	0	0	0	0	0	0
	Total	38	100	66	100	104	100
Relationship to household	Head	32	84.2	4	6.1	36	34.6
	Son/daughter	6	15.8	39	59.1	45	43.3
	Husband/wife	0	0	13	19.7	13	12.5
	Other	0	0	10	15.1	10	9.6
	Total	38	100	66	100	104	100
Size of household	1-3	3	7.9	6	9.1	9	8.7
	4-6	5	13.2	14	21.2	19	18.3
	7-9	8	21.1	10	15.2	18	17.3
	10 and above	22	57.9	36	54.6	58	55.8
	Total	38	100	66	100	104	100

The median total direct cost was N8,245 (US$51)(range N3,420-N29,120) for all visits, including costs for escorts (Table 2). Total median direct costs were N9,020 (US$56) for men and N7,520 (US$47) for women (p < 0.09). The median direct non-medical cost for all participants was N4,050 (US$25) which is 49% of total direct costs (Table 3). The median cost of travel was considerably lower for those living within 20 km of the hospital (N375, US$2) than those living at a distance of more than 20 kms (N 4,200, US$ 26) (p < 0.001). Transport accounted for 75% of all direct non-medical costs.

**Table 2:** **Total direct patient cost for cataract surgery (values in USD**)

**Details of costs for each visit**		**Male (n = 38)**		**Female (n = 66)**		**All (n = 104)**	
		**Median**	**Range**	**Median**	**Range**	**Median**	**Range**
Assessment visit	Cost for escort*	4.5	0 - 24.8	3.7	0 - 37.3	4.3	0 – 37.3
	Meal cost	0.6	0 – 7.5	0.6	0 – 11.2	0.6	0 – 11.2
	Travel cost participant	3.7	0 – 18.6	2.8	0 – 12.4	3.1	0 – 18.6
	Accommodation	-	-	-	-	-	-
	Hospital charges	1.4	0.1 – 18.8	1.4	0.1 – 6.3	1.4	0.1 – 18.8
	Subtotal is	10.7	1.4 – 51.1	9.1	1.4 – 53.5	10.1	1.4 – 53.5
Surgery visit	Cost for escort*	5.1	0 - 24.8	3.7	0 – 37.3	4.3	0 – 37.3
	Meal cost	0.9	0 – 8.4	0.6	0 – 22.4	0.6	0 – 22.4
	Travel cost participant	3.7	0 – 18.6	2.5	0 – 12.4	3.1	0 – 18.6
	Accommodation	-	-	-	-	-	-
	Hospital charges	19.9	18.3 – 36.6	19.9	17.7 – 32.3	19.9	17.7 – 36.6
	Subtotal	33.5	19.9 – 70.8	28.9	19.9 – 85.1	30.0	19.9 – 85.1
Follow up visit	Cost for escort*	4.2	0 – 28.6	3.7	0 – 25.5	3.9	0 – 28.6
	Meal cost	0.3	0 – 11.2	0.6	0 – 5.6	0.6	0 – 11.2
	Travel cost participant	3.7	0 – 18.6	2.8	0 -12.4	3.1	0 – 18.6
	Accommodation	-	-	-	-	-	-
	Hospital charges	3.1	3.1 – 6.8	3.1	0 – 5.0	3.1	0 – 6.8
	Subtotal	11.8	3.1 – 60.2	10.2	0 – 41.3	10.9	0 – 60.2
	TOTAL	56.0	24.3 – 180.9	46.7	21.2 – 141.4	51.2	21.2 – 180.9

*Cost of escort includes costs for his meals, transport and accommodation for all the visits.

Note: US$1 = N161.

**Table 3:** **Non-medical direct costs and total patient costs (values in USD**)

**Variables**	**N**	**Total direct costs**		**Direct medical costs**		**Direct non-medical costs**		
				**Amount**		**Amount**		**Total**
		**Median**	**Range**	**Median**	**Range**	**Median**	**Range**	**%**
**Sex**								
Male	38	56.0	24.3 - 180.9	25.3	22.8 - 41.7	27.3	0 - 156.5	49
Female	66	46.7	21.2 - 141.4	24.3	21.2 - 36.8	22.4	0 - 114.3	48
**Age group**								
Less than 65	66	47.3	21.2 - 180.9	24.3	21.2 - 41.7	22.4	0 - 156.5	47
65 or older	38	62.2	24.3 - 152.3	25.3	22.8 - 41.4	33.1	0 - 115.5	53
**Marital status**								
Married	79	49.2	21.2 - 180.9	24.3	21.2 - 41.7	24.8	0 - 156.5	51
Not married	25	54.2	24.3 - 126.8	25.3	24.3 - 30.6	29.8	0 - 89.4	55
**Distance**								
Near	60	40.1	21.2 - 114.4	24.3	21.2 - 41.7	9.9	0 - 91.3	25
Far	44	84.1	27.5 - 180.9	25.7	22.2 - 36.8	58.4	3.1 - 156.5	69
**Socioeconomic status**								
0-1 assets	36	42.7	21.2 - 126.8	24.3	21.2 - 27.5	18.3	0 - 99.4	43
2 or more assets	68	56.0	24.3 - 180.9	25.7	22.2 - 41.7	29.2	0 - 156.5	52
**Household status**								
Household head	36	52.3	24.7 - 180.9	25.9	22.8 - 41.7	26.1	0 - 156.5	50
Other	68	48.0	21.2 - 141.4	24.3	21.2 - 36.8	23.3	0 - 114.3	49
**ALL**	**104**	**51.2**	**21.2 - 180.9**	**24.3**	**21.2 - 41.7**	**25.2**	**0 - 156.5**	**49**

Distance = Distance from place of residence to hospital.

For all visits, the median total direct cost was N2,025 (US$13) for escorts and N6,320 (US$39) for participants (p < 0.01) (Tables 4 and 5). Costs incurred for escorts accounted for 37% of all costs, being similar for males and females (median N 2,100; US$13 and N1,875; US$12 respectively). Most men (92%) already had money to pay for the services and the remaining 8% sold possessions. More women than men sold assets or took out a loan (13.6% vs 7.9% respectively): 7% of married women sold assets or took out a loan compared with 20% of unmarried women.

**Table 4:** **Costs incurred by escort and the patient (values in USD**)

	**Male**		**Female**		**All**	
**(All visits)**	**Median**	**Range**	**Median**	**Range**	**Median**	**Range**
Patient only (p = 0.02)	42.4	24.3 - 102.6	37.5	21.2 - 77.1	39.3	21.2-102.6
Escort only (p = 0.3)	13.0	0 - 78.3	11.6	0 - 8 2.0	12.6	0 - 82.0

Note: US$1 = N161.

**Table 5:** **Total patient direct costs for different components in all visits (values in USD**)

	**Male**		**Female**		**All**	
	**Median**	**Range**	**Median**	**Range**	**Median**	**Range**
Meals	2.2	0 - 26.1	1.9	0 - 23.6	1.9	0 - 26.1
Travel	11.2	0 - 55.9	8.4	0 - 37.3	9.3	0 - 55.9
Accommodation	-	-	-	-	-	-
Hospital charges	25.3	22.8 - 41.7	24.3	21.2 - 36.8	24.3	21.2 - 41.7
Total	**42.4**	**24.3 - 102.6**	**37.5**	**21.2 - 77.1**	**39.3**	**21.2 - 102.6**

Note: US$1 = N161.

## Discussion

A limitation of the study was that the desired sample size was not reached. This was because the study was undertaken during the rainy season when transport is difficult meaning that fewer cataract surgeries were performed. A larger sample may have been adequately powered to detect statistically significant differences in costs between men and women. Another limitation was that productivity costs (e.g. loss of wages) were not assessed. In subsistence farming communities, as in Zamfara State, time spent away from agricultural activities may also have economic consequences which would add to the financial barriers.

In this study more women than men had recently accessed cataract surgery despite several studies indicating that men usually outnumber women. This is reflected in the higher cataract surgical coverage in males reported in studies in other States in Nigeria [3,7] and in the national blindness survey (men 51%, women 30%) [6]. Our findings probably reflect the timing of the study as men are busy farming during the rainy season whereas women are busier during the harvest. As direct costs were somewhat higher for men than for women, overall patient costs are likely to have been underestimated. Further studies to measure patient costs or to assess the impact of interventions to minimise cost in Africa will need to take seasonal patterns of health seeking behaviour into account.

The gender difference in costs were as anticipated, reflecting gender differences in social roles, as most women in northern Nigeria have little income to spare for healthcare and have little or no influence on household spending [3], as decisions are made by household heads. In our study the impact of costs was greatest in unmarried women as one in five had to sell possessions or take out a loan.

The only other study of patient costs for cataract surgery was undertaken in a tertiary level, faith based eye hospital in urban Zambia [15], but this study did not include escorts’ costs. Patient costs in Zambia were considerably higher (US$151) than in our study (US$51) as income from user fees had to cover the majority of the provider’s costs [15] whereas Gusau Eye Clinic is a government facility.

Direct non-medical costs accounted for 49% of the total direct costs, most being for transport, reflecting the long distances patients and their escorts travel, usually in private taxis or buses. There were no accommodation cost as patients and their escort wait on hospital premises overnight to reduce costs. In many instances participants did not need an escort after surgery on account of good visual outcomes.

The median total direct patient cost of cataract surgery (US$51) represents at least 50 days income for 70% of the population in Zamfara State. A study in Nigeria also found that most glaucoma patients cannot afford treatment as more than half of their monthly income would be spent on care and medication [17]. Other studies on non-eye health services have revealed expenditure that is even higher, being beyond annual household income [18]. In our study most families already had enough money for the services, which suggests that it is the wealthier in the community who are accessing services, as has been shown in other studies [19,20]. There is now an extensive body of literature on the impact of user fees on the interrelated factors of equity, access and quality of services [21]. What is clear is that lowering or increasing user fees has an immediate impact on access as was reported for maternal and child health care in Ethiopia [20]. The subsidy provided by the international non-government organization contributed to an annual increase in CSR and it has been estimated that access would have been 70% lower without the subsidy.

One of the outcomes of a good health system is that it protects communities from financial risk [22]. Protection can result from any mechanism which reduces patient costs, such as health insurance, or performance based financing. However, there is evidence that removal of user fees alone does not give sustained increase in access unless policies are in place to address lack of trained staff. The Nigerian government is committed to universal health coverage with ongoing support to primary health care and infectious and non-communicable diseases. There are also plans to provide universal access to cataract surgery, which, if implemented, should reduce inequity of access, by reducing direct medical costs.

This study is the first of its kind to analyse costs of cataract surgery to patients and their families in rural Africa and detailed information was collected for a number of hospital visits over a short period of recall. The findings that direct medical costs were consistent shows accuracy of recall, which is likely to extend to recall of direct non-medical costs.

## Conclusions

These findings clearly indicate the need for innovative approaches by policy makers and programme planners to make eye care services more accessible. In Zamfara, one way to reduce non-medical costs would be to provide transport. Many eye care programmes in low and middle income settings do not meet the World Health Organisation recommended CSR target of 2000, being only 500 in Zamfara State despite the subsidy and high quality services. There is an urgent need for research into interventions which reduce the cost of cataract surgery to patients and their families in rural Africa, particularly for the most vulnerable such as unmarried women. Interventions could include higher subsidies or a voucher system for the most vulnerable, coupled with free transport.

## Abbreviations

CSR: Cataract surgical rate
N: Nigerian naira
